# Loss of resilience preceded transformations of pre-Hispanic Pueblo societies

**DOI:** 10.1073/pnas.2024397118

**Published:** 2021-04-28

**Authors:** Marten Scheffer, Egbert H. van Nes, Darcy Bird, R. Kyle Bocinsky, Timothy A. Kohler

**Affiliations:** ^a^Environmental Science Department, Wageningen University, 6700 AA Wageningen, The Netherlands;; ^b^Department of Anthropology, Washington State University, Pullman, WA 99164-4910;; ^c^Crow Canyon Archaeological Center, Cortez, CO 81321;; ^d^Montana Climate Office, W.A. Franke College of Forestry and Conservation, University of Montana, Missoula, MT 59812;; ^e^Santa Fe Institute, Santa Fe, NM 87501

**Keywords:** archaeology, climate change, resilience, collapse

## Abstract

Climate extremes are thought to have triggered large-scale transformations of various ancient societies, but they rarely seem to be the sole cause. It has been hypothesized that slow internal developments often made societies less resilient over time, setting them up for collapse. Here, we provide quantitative evidence for this idea. We use annual-resolution time series of building activity to demonstrate that repeated dramatic transformations of Pueblo cultures in the pre-Hispanic US Southwest were preceded by signals of critical slowing down, a dynamic hallmark of fragility. Declining stability of the status quo is consistent with archaeological evidence for increasing violence and in some cases, increasing wealth inequality toward the end of these periods. Our work thus supports the view that the cumulative impact of gradual processes may make societies more vulnerable through time, elevating the likelihood that a perturbation will trigger a large-scale transformation that includes radically rejecting the status quo and seeking alternative pathways.

## Significance

Collapse of civilizations remains one of the most enigmatic phenomena in human history. In this paper we provide quantitative evidence that loss of resilience systematically preceded collapses. We take advantage of unique time series documenting both construction activity and climate conditions for pre-Columbian societies of the southwestern United States on an annual basis over a period of eight centuries. These data cover five transformations encompassing shifts to novel constellations of beliefs, social practices, and styles of art and architecture. The remarkable high-resolution time series allowed us to quantify the dynamics of social fragility using numerical techniques for probing resilience. Our results demonstrate that all but one of these transformations happened after decades of rising social instability.

Explaining the spectacular collapses and transformations observed in prehistoric civilizations remains difficult. An obvious approach is to search for external perturbations that can be held responsible. The possibility of an adverse climatic turn has received particular attention (1–8). On the other hand, it has been argued that societal tensions may rise as populations approach carrying capacity (9), wealth becomes concentrated in the hands of a small elite (10, 11), or the costs of rigidity (12) and complexity (13) take their toll. Interactions between these and other mechanisms may gradually dismantle societies, but evidence for such systemic destabilization is hard to obtain. In principle this hypothesis is best tested in prehistory, where many cycles of societal aging, collapse, or transformation are completed. Yet, in such settings, low temporal resolution typically weakens attribution of causation (14).

Here, we address this classical problem using tree-ring series representing, at annual resolution, both construction activity and paleoclimates in a substantial portion of the pre-Hispanic US Southwest (for a map see figure 1 of ref. 15). The regional prehistory is known for its periodic discontinuities leading to a succession of marked periods each characterized by a constellation of architecture, ceramics, and settlement configurations distinct enough that early researchers could identify the temporal succession even before they had absolute dating methods (16). Analysis of tree-ring-dated wood recovered from archaeological sites eventually revealed that construction activity in this region was clustered in four periods, sharply separated by intervals of low activity (17). These periods of high activity (reflected in modes in the histograms of counts of wood-cutting dates through time) match four distinct periods, identified in 1927 by archaeologists as Pecos periods: the Basketmaker III (BMIII) and the Pueblo I to III (PI, PII, and PIII). The first study to identify these marked peaks and valleys in tree-ring dates proposed that periods of peak cutting corresponded to good maize farming conditions separated by droughts (17). However, a more recent study, using a larger set of tree-ring dates and highly localized reconstructions of temperature, precipitation, and tree-cutting activity, shows that on average the periods of high construction were no better for maize production than the periods of low construction, though they terminated in productive downturns that ranged from mild to severe (15). The troughs in construction activity coincided with community disaggregation at both local and subregional levels, as well as material and technological changes visible in the archaeological record.

What could have driven those societies to abruptly abandon established ways of doing things and start experimenting with novel settlement locations, architecture, and pottery styles? Here, we examine whether a loss of resilience, making the status quo increasingly fragile, may have brought those societies toward a tipping point due to the accumulation of internal processes as they aged. We first present a conceptual framework for how this could work and explain how such loss of resilience may be detected in data. We then demonstrate evidence of declining resilience in reconstructed time series of construction activity in these Pueblo societies, and finally we discuss how results compare to archaeological findings.

## A Theory for the Loss of Resilience of the Status Quo and Its Dynamical Indicators

**Fig. 1.**
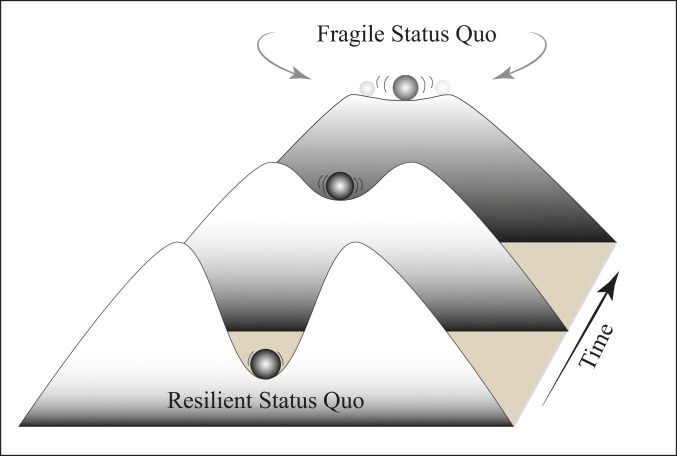
Conceptual model of intrinsic loss of resilience of the status quo in aging societies. Processes such as population growth leading to shortages may increase violence and other problems, reducing the attractiveness of the status quo (the way of doing things anchored in social practices and rituals). In the resulting fragile state, returns to normal upon perturbations such as droughts become increasingly slow, and the likelihood that the system flips out of the societal basin of attraction rises. A mathematical model is presented in *SI Appendix*.

The collapse of ancient societies may entail marked population declines if famine or violence are taking a toll (18). However, the discontinuities inferred from archaeological work may often reflect societal transformations rather than population collapses (19). Radical transitions may be driven by collapse of trust in the old way of doing things, including its rituals and social and physical structures. Such distrust can be self-propagating, cascading contagiously through the society (20). In dynamical systems theory terminology, the status quo in a society may be seen as an attractor, i.e., a state with stabilizing feedbacks (21). One obvious stabilizing force is the vested interest of an elite who benefits from the status quo and uses her capital to maintain it. A second stabilizer is the so-called “sunk-cost effect.” This is a reluctance to abandon things in which much has been invested, even if it would be rationally better to abandon them (22). In societies, this tends to hold not just for physical structures in villages, and landesque capital, but also for rituals and beliefs (9). A third ubiquitous stabilizing mechanism is contagiousness of attitude and opinion, making it difficult to deviate from the view held by the majority, and therefore stabilizing the status quo (20). Despite such stabilizing mechanisms, radical change occasionally happens in societies. In the view we propose here, societies become ready for such change when the old way of doing things becomes less attractive (23). This means that the attractor of the status quo becomes less resilient, which may be visualized in a classical way as the basin of attraction becoming smaller and shallower (Fig. 1).

This raises the question of whether such loss of resilience may be objectively (and materially) detected in some way. It turns out that across many dynamical systems, declining resilience in the vicinity of a tipping point may lead to subtle but observable changes in the nature of fluctuations, due to a generic phenomenon known as “critical slowing down” (24, 25), referring to the fact that recovery upon a perturbation becomes slow. In our graphical model (Fig. 1) this can be intuitively understood as the ball rolling more slowly down the shallow slope than the steep slope. In a system with stochastic perturbations (such as random climate shocks), the effect of such slowing down is that the “memory” in the state increases, thereby resulting in rising temporal autocorrelation and variance (25–28). This phenomenon is generic in the sense that it is independent of the idiosyncrasies of the system involved and has been found to happen prior to critical transitions in complex systems ranging from the human body (29, 30) to climate elements (31, 32) and ecosystems (27, 33).

To illustrate how this could play out for social unrest we use a dynamical model of abandonment of the status quo in a society (20, 34, 35) (Box 1 and *SI Appendix*). The equation captures the mean attitude toward the status quo in a society consisting of numerous individuals, each one somewhat different from the others. For each person, the attitude toward the need for change is −1 if she is content with the status quo and +1 if she is discontent and wants things to change. All individuals determine their attitude toward the need for change depending on their own perceptions of the seriousness of problems such as food insecurity, unfair inequality, and the risk of uncontrolled violence. Importantly, they also have a tendency to adhere to the attitude of their peers. If this tendency is sufficiently large [and in fact pre-Hispanic Pueblo societies have been characterized as highly conformist, particularly in the second millennium AD (36)], the model predicts classical hysteresis and tipping points in attitude (20). When we drive this model toward a tipping point by gradually increasing the perceived seriousness of a problem, temporal autocorrelation and variance of the fluctuations in attitude rise prior to a massive transition in attitude. This is not something specific to the model we use here for illustration. Rising variance and autocorrelation are generic indicators of loss of resilience reflected in a phenomenon known as critical slowing down (24, 25, 37, 38). Loosely, in our case one may think of this as resulting from the fact that the return rate of attitude back to normal upon a perturbation (such as a drought-induced harvest failure) is slower when the society is closer to the tipping point—where approval of the status quo is less resilient. In that case, any social unrest will take longer to fade away, leaving the attitude more correlated to that in the previous year.

Box 1 Indicators of critical slowing down in a model of social attitude.
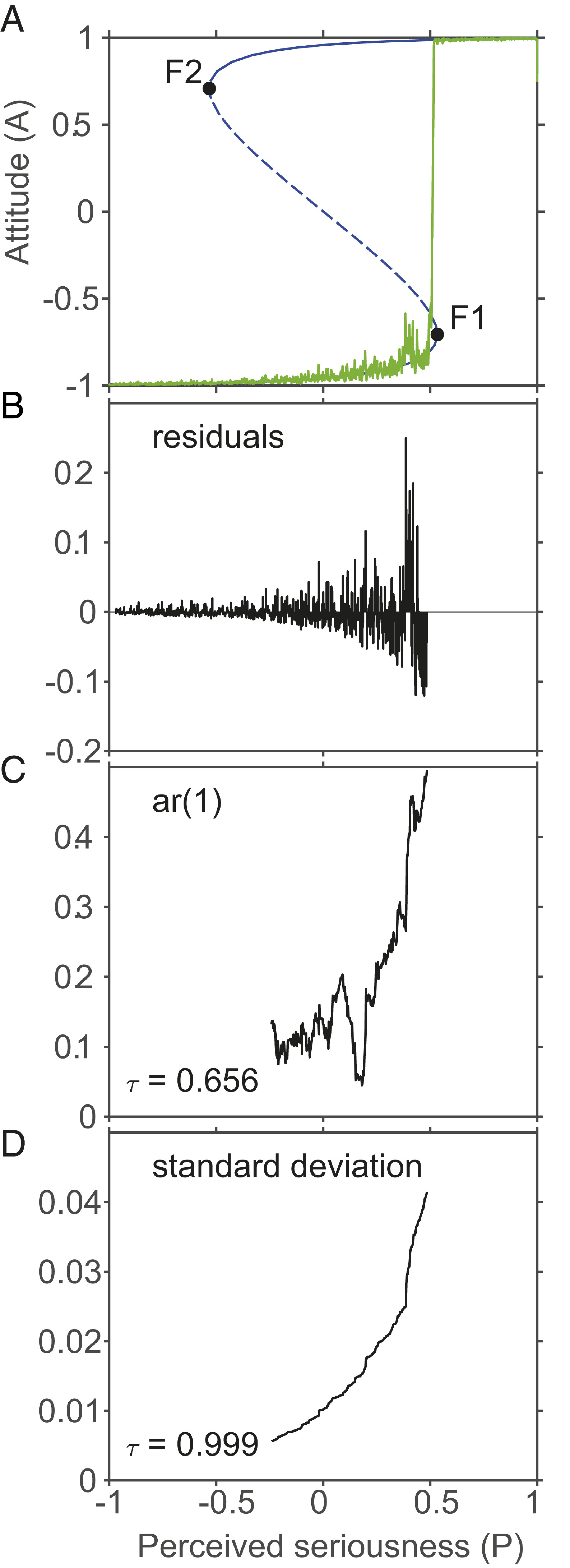

**Box 1 Figure.** (*A*) Evolution of mean attitude (A) toward the need for change, depending on the perceived seriousness of problems (P) such as food insecurity or violence, and on social pressure to adhere to the same attitude, as a group approaches a transition (F1). (*B*) The detrended fluctuations in attitude from *A*, which rise prior to the transition. (*C*) The temporal autocorrelation (AR1) of the detrended fluctuations in attitude from *B*. (*D*) The standard deviation of the detrended fluctuations in attitude from *B*. Kendall’s rank correlation (τ) of the autocorrelation and standard deviation with time shown in *C* and *D*.

## Inferring Resilience from Construction Dynamics in Pueblo Societies

**Fig. 2.**
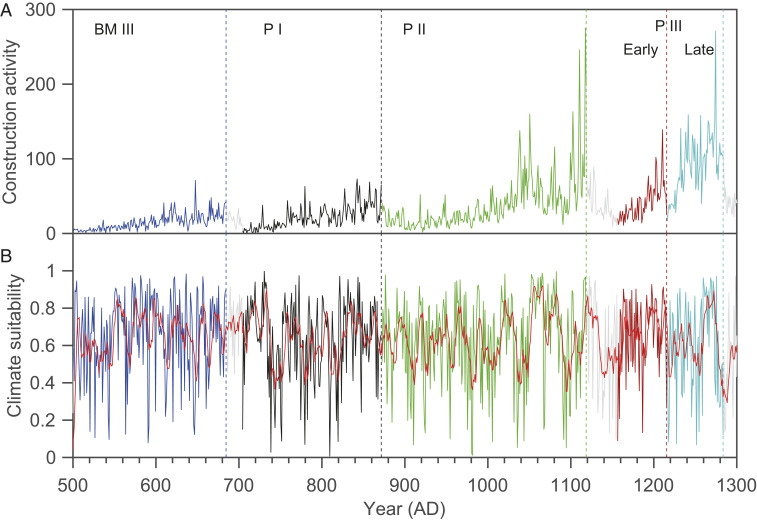
Dynamics of construction activity and climate suitability in pre-Hispanic Pueblo societies. (*A*) Number of trees felled in any year as a proxy for construction activity. (*B*) Fraction of the area with climatic conditions conducive to successful maize dry farming estimated using temperature and precipitation inferred from tree-ring data (15). The red line represents the average climate suitability over the preceding 6 y. Vertical dashed lines indicate the onset of “collapses” inferred from episodes of dwindling construction activity (gray parts of the dynamics) corresponding closely to terminations of recognized cultural periods [BMIII, PI, PII, and PIII (15)]. Colored parts mark the phases of growing construction activity over which we scanned for indicators of changing resilience (Fig. 3).

We cannot measure attitudes in ancient societies, but we might infer their dynamics indirectly through archaeological materials. The idea is that change in attitudes toward the prevailing social system may be reflected in change of activities that leave traces in the archaeological remains. To explore this possibility, we take advantage of the extraordinary long and high-resolution time series of Puebloan tree-cutting activity (Fig. 2). The wood in these series comes from structural elements of settlements recovered by archaeologists over a century of investigation. Tree rings in this wood record both the year in which a tree was felled (where the outermost ring is preserved) and also the local climatic fluctuations during the life of the tree [although ordinarily the climatic information is not inferred from wood recovered from archaeological sites (39)]. Obviously, tree-cutting activity cannot be seen as a straightforward indicator of attitude toward the status quo. Nonetheless, it seems likely that factors such as perceived insecurity, confidence in the future, uncertainty, and social unrest would in some way or another affect the business-as-usual cutting of trees for construction. Therefore, variance and temporal autocorrelation in tree cutting (or any other social activity) will in part reflect variance and autocorrelation in social attitudes and sentiments.

**Fig. 3.**
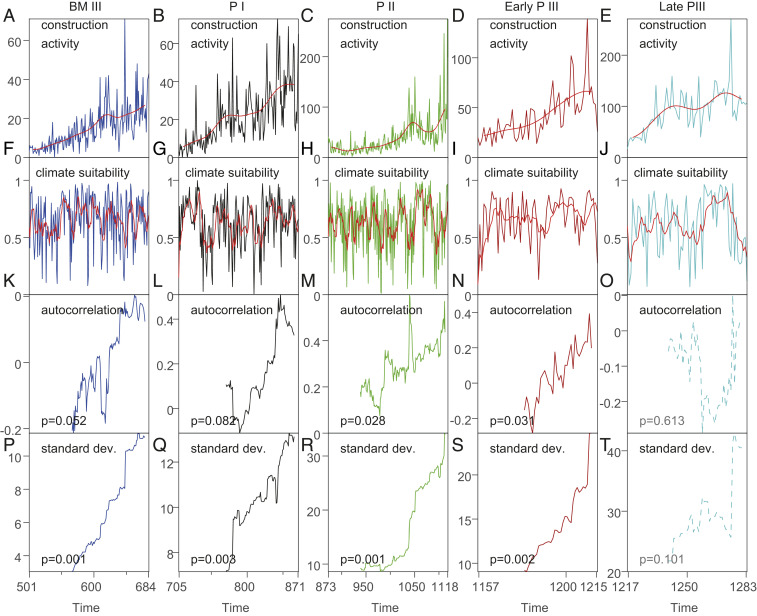
Proxies for social and climatic dynamics leading up to collapse of construction activity in each of the archaeologically distinct periods. Note that the PIII period was divided into Early and Late PIII (see the main text and *SI Appendix*, Fig. S4 for the entire PIII). The periods correspond to those color-coded in Fig. 2. (*A*–*E*) Construction activity inferred from number of trees felled (as in Fig. 2). The datasets are plotted together with the smooth curve used for detrending before computing the indicators shown in the other panels (see *SI Appendix* for methods). (*F*–*J*) Reconstructed proportion of the study area suitable for dry farming of maize (15). The smoother red curves represent climate suitability averaged over the past 6 y. (*K*–*O*) Slowness of dynamics in construction activity indicated by the lag-1 autocorrelation AR(1). Dashed lines indicate high *P* values (*P* > 0.1). (*P*–*T*) SD of fluctuations in construction activity.

We checked for indicators of slowing down in this variable by scanning for temporal autocorrelation in a moving window over each of the classically recognized periods (*Materials and Methods*). The results reveal a marked rise in the temporal autocorrelation of tree-cutting activity over the lifetime of BMIII, PI, PII, and early PIII, suggesting loss of resilience as societies approached a tipping point for transformation (Fig. 3 *K*–*O*). As our model analysis illustrates, time-correlated randomness may produce trends as well as quite irregular patterns in the short time series we have at hand (Box 1 and *SI Appendix*, section I).

To estimate the probability that the rise in autocorrelation in this set of observations is due to chance, we generated 1,000 randomized time series with the same frequency structure as the original data and compared the observed tau values to the distribution of tau values in the randomized time series (see *Materials and Methods* for details). It turns out that the estimated probability of this series of rising temporal autocorrelations occurring by chance is small (*P* = 0.0038; *SI Appendix*, Table S1). This result is robust against methodological choices such as data smoothing, the size of the moving window used for the computation of correlation, and the kernel width used for detrending (*SI Appendix*, Figs. S5 and S8). Note that the lag-1 autocorrelation is occasionally negative, especially at the beginning of periods. This suggests a tendency for an alternation of years with higher and lower tree-felling activity in a 2-y cycle. To check how this might influence our results, we applied a 2-y moving average and repeated all analyses. This removed the negative autocorrelation but left all results qualitatively the same (*SI Appendix*, section III and Figs. S9 and S10). In all periods we also find a marked increase of variance in the detrended fluctuations (Fig. 3 *P*–*T*) consistent with critical slowing down (28). By itself, variance is a less robust indicator than temporal autocorrelation, but simultaneous rising trends in both indicators strongly suggest the occurrence of critical slowing down (40), pointing to a loss of resilience as those societies approached the end of a period.

The pattern in the late PIII period, however, is different from what we see in the other periods (see *SI Appendix*, Fig. S4 for a detailed analysis of PIII). Autocorrelation and variance do rise over the early PIII period, but then there is a sharp drop of construction activity marking the start of the late PIII period (at 1217 CE). This transition does not correspond to the onset of a new Pecos period but nonetheless involves a marked change in construction activity. The abrupt drop in tree felling is followed by about a decade of constant anomalously low activity (Fig. 2 and *SI Appendix*, Fig. S4). Trends in both autocorrelation and variance over the late PIII period are erratic. One possibility is that those are simply statistical vagaries due to noisy data. After all, the entire PIII period is short relative to the other periods and splitting it into subperiods further reduces statistical power (*SI Appendix*). However, as we argue below, there is also the possibility that the change during late PIII was fundamentally different from what happened in any of the other periods.

## Probing the Role of Climatic Variability

In these Pueblo societies most of the diet consisted of maize (41, 42). Maize productivity varied greatly from year to year depending partly on temperature but mostly on precipitation variability (15). To examine the potential role of climate in driving social dynamics, we analyzed how fluctuations in tree-cutting activity relate to annual variation in climate characteristics relevant for maize production (Fig. 2). The climate indicator we used is the fraction of the study area with conditions conducive to successful maize harvests as inferred from tree-ring data sensitive to local variability in temperature and precipitation (15). Overall, variations in construction activity were correlated to the suitability of climatic conditions for maize production averaged over (the preceding) 6 y (*SI Appendix*, Table S1), especially when using a 2-y averaging to remove the biannual cycle tendency in tree-cutting activity (*SI Appendix*, Table S2).

However, a look at the patterns of maize productivity over the different periods immediately reveals that the societal transformations were not simply driven by climate downturns (Figs. 2 and 3 *F*–*J*; see also ref. 43). Marked dips in maize productivity occur throughout all periods without leading to collapse. Although BMIII and PI do indeed terminate in times of low productivity, these downturns were not as extreme as some earlier episodes which did not coincide with a period termination. At the end of PII, construction activity declines before the mid-1100s drought. A correlation between some of the social transitions with episodes of poor maize productivity suggested by earlier work (15) was based on kernel-smoothed production estimates, thus involving conditions for maize productivity over the past years but also the coming years around any data point. Instead, we focus on climate conditions over the recent past (the current year and the five preceding years; see smoothed curves in climate suitability panels of our figures), since, logically, social dynamics cannot be driven by future climatic conditions.

Things are different, however, for the PIII period. The early-to-late PIII transition coincides with years of unusually poor growing conditions for maize (44) (Fig. 2; for more detail see *SI Appendix*, Fig. S4 *C* and *F*). Late PIII, however, is characterized by improved maize growing conditions (Fig. 3*J*), even as the farming population had presumably begun to leave the northern portions of this area. In the southern portion, populations began to spatially contract, meaning the proportion of occupied land within the study area was decreasing. The final collapse of building activity beginning in the 1270s was indeed accompanied by a strong climatic downturn (Figs. 2 and 3 *E* and *J*). Finally, the termination of PIII coincides with the emigration of the entire remaining farming population from the Four Corners toward destinations to the south and southeast (45). Together with the anomalous patterns of autocorrelation in building activity, this suggests that the famous and dramatic end of late PIII was indeed unlike any of the earlier transitions in Pueblo society.

## Discussion

Our results suggest that rising social fragility led to most of the repeated transformations of Puebloan societies in the American Southwest between AD 500 and 1300. Increasing temporal autocorrelation together with rising variance is a tell-tale sign of critical slowing down associated to loss of resilience (25, 27, 28). Also, independent evidence for increasing violence toward the end of PI, PII, and PIII (46) is consistent with our theory of dwindling resilience of the business-as-usual situation. If our diagnosis is correct, and these societies became increasingly fragile over time, what could be the mechanisms behind that repeated pattern? Although reconstructions necessarily remain speculative in part, other archaeological data and climate reconstructions make it possible to fill in more of what actually may have happened (9, 15, 46).

For instance, piecing together diverse lines of evidence Bocinsky et al. (15) have hypothesized that the cyclic pattern of construction activity in these societies may represent alternate phases of exploration and exploitation. During the exploration phases, populations spread out more on the landscape and may have experimented with new locations and social forms. In such periods, some practices and places likely proved to work particularly well. Those ways of doing things may have expanded, establishing the status quo during the subsequent period of exploitation. In exploitation phases, successful clusters likely grew from both internal demographic processes and immigration to these attractive areas. Cultural transmission and emulation along with these population movements likely increased stylistic conformity across the region. Maize farming and other activities were productive, while social and ritual practices were tuned to successfully stabilize the system.

While this reconstruction seems plausible, it is precisely during periods of seemingly thriving exploitation that we find evidence for critical slowing down. Why did their apparent success undermine resilience over time? One possibility is that the rising temporal autocorrelation should be read as an increasing tendency for people to simply do what they did last year, rather than what would be most appropriate given current climatic conditions. However, this model of increasing unresponsiveness to environmental conditions would likely not result in the increasing variances that we also see in these sequences. More to the point, most periods evidence increasing and positive cross-correlations between tree-cutting activity and the climatically controlled size of the maize-growing niche (*SI Appendix*, Tables S1 and S2) (though only one of these is statistically significant). So, we see little evidence for this hypothesis.

Other possibilities deal with processes that would be expected to increase over the life of villages in this area as they increased in size. Local growth and aggregation at the village level could lead to shortages as per-capita production decreased and accessing increasingly distant fields became harder (47). Rising inequality during periods of exploitation may have contributed to tensions, as small initial differences in quality and quantity of lands held and workforce sizes in each lineage occupying particular villages led to differential wealth accumulation across lineages. Indeed, for the VEPIIN region of southwestern Colorado there is evidence for peaks in wealth differences in late PI, late PII, and early PIII followed in each case by peaks in violence (48) coinciding with poor climates for maize production, which may have been their proximal cause (47). The overall image might be one in which declining per-capita yields, increased inefficiency in access to fields, depletion of slowly renewing resources near large villages, increasing inequality, and perhaps mounting disease under aggregation (49)—all demonstrable except the last—tended to reduce resilience of the acceptance of business-as-usual social dynamics. Food shortages, resentments, and outbreaks of violence are logical outcomes under this scenario, further delegitimizing the practices and rituals that tied people and places together, as well as trust in local leadership. Violence would have been particularly deleterious since it would simultaneously inhibit access to more remote fields while also promoting further aggregation for security (50). As resilience of acceptance of the status quo declined such outbreaks may have taken longer to fade away, effectively corresponding to critical slowing down as reflected in rising autocorrelation and variance of sentiments and deviations from the normal pattern of activities. It is likely that the transformations that eventually happened were overdue, as the psychological “sunk-cost effect” of the physical structures and entire set of beliefs, rituals and social practices would have discouraged people from abandoning the established way of doing things, even if it would rationally have been the best thing to do for some time already (9). Eventually, however, an unstoppable contagious shift of attitude (as in our abstract minimal model) may have cascaded through these societies, leading to radical abandonment of the status quo.

Not all transitions were the same, however. The most dramatic took place at the end of PIII, with massive migrations and abandonment of many practices, some of which had been prevalent for centuries (51). Our findings underline its distinct character. Although climatic conditions for maize cultivation temporarily improved (and autocorrelation dropped, which suggests growing resilience in this brief episode), the emigration that terminated PIII was accompanied by a strong climatic downturn. It thus seems that the final move out of the region was of a different character altogether than the series of transitions that preceded it. Possibly, societies were sensitized already by the sharp drought marking the end of early PIII followed by a decade of anomalously low building activity. Notably, large-scale immigration during the early-to-late PIII transition (around 1220), possible pressure from foraging groups (52), and the attraction to a new way of life forming in the northern Rio Grande and elsewhere all seem to have decisively changed the dynamics in ways we do not yet fully understand (see also ref. 53). In any case, this grand final transition was different from anything that happened before.

Most discussions of “collapses” and climate in archaeology suffer from rather broadly dated paleoclimatic proxies, or proxies with uncertain effects on society, or broadly dated archaeological periods—or all three—making it difficult to obtain “robust and nuanced causal interpretations about the links between human societies and their environment” (54). Here we profit from a paleoclimatic proxy that is directly linked to agricultural production and a behavioral proxy that is directly linked to construction activity, both having annual resolution. Such unambiguous temporal linkage between climate and social action cannot always be obtained even in the historical record. We find that some Puebloan transformations that seemed linked to climate have an important social component, but we also conclude that the famous final depopulation of the northern Southwest lacks those internal social early warning signs visible in earlier transitions and therefore must be attributed to external factors including climatic variability and external social pressures. Clearly the relationship between climatic variability and social success must be analyzed with care.

In conclusion, our results resonate with archaeological evidence that successful Puebloan societies repeatedly lost resilience in a persistent pattern until at least the early 1200s. Such loss of resilience implies that eventually even minor adverse events could trigger a societal transformation. In this record, social dynamics typically determine which climatic events or other perturbations will precipitate reorganization. This pattern may be common in other societies, and this may be one reason why it is often difficult to find clear correlations between climatic perturbations and transformations (see also ref. 55). Signs of critical slowing down have also been found in Neolithic European dynamics before regional collapses were observed that had no obvious relationship to climate conditions (56, 57). Even premodern states in which “good governments” created a social environment providing benefits to citizens were subject to collapse when “eventual moral lapses in the central leadership brought loss of citizen confidence, decline in fiscal health and government services, demographic decline, growing inability of the central authorities to control crime and administrative corruption, and the eventual rise of opposition movements and political polarization” (58), not unlike the conditions we suspect toward the end of three of our periods of exploitation. This does not mean that major climatic extremes cannot play a decisive role in driving societies to collapse or reorganize—as they seem to have had among Pueblo societies in the mid-late 1200s—but the interplay of such external forcing with the internal social dynamics is critical to understand. Here we provide an avenue for doing precisely that. A climate event such as a prolonged drought may trigger a critical transition toward abandoning the old way of doing things, but the chances for this to happen will be larger if there is already a latent discontent due to growing constraints on success such as inequality, extreme aggregation, violence, or other factors that may slowly accumulate through multiple generations.

## Materials and Methods

Our time series consists of yearly counts of the wood-cutting dates found on archaeological sites (15). The results presented in the main text occasionally show some negative lag-1 autocorrelation, especially at the beginning of periods. This appears to signal a tendency for an alternation of years with higher and lower tree-felling activity in a 2-y cycle. To check how this might influence our results we removed this negative autocorrelation using a 2-y moving average and repeated all analyses. As shown in *SI Appendix*, section III and Figs. S9 and S10 all main results remain qualitatively similar. The periods of high tree-cutting activity were followed by collapses of activity. The trends in resilience indicators before each of these collapses were determined using the method of Dakos et al. (32). This method is designed to test the hypothesis that the resilience gradually decreased before the collapse. We first determined the time of collapse in each of the regimes by visual inspection. The resulting six time series were detrended with a Gaussian kernel smoothing algorithm with a default bandwidth of 30 y. This detrending is needed as a change in trend can cause a change in autocorrelation and variance which is not related to critical slowing down (38). The detrended data were further analyzed using a sliding window. We used 60 y as the default size of this window. As the time series for the early- and the late-PIII period are much shorter than the other periods we had to use a smaller bandwidth for the detrending kernel (15 y) and a smaller size of the moving window (20 y). As results depend on such choices we analyzed the robustness of the results for a broad range of bandwidth and window sizes (*SI Appendix*, Figs. S5, S7, S8, and S10).

The lag-1 autocorrelation and SD of the detrended data are computed for each position of the moving window and plotted at the last year of the window. Thus, those indicators characterize the period preceding the data point. This is important to make them useful for the analysis of what happens preceding a collapse (38). For the same reason we excluded the data of the collapses themselves. The lag-1 autocorrelation was determined by fitting a one-variable autoregression model. The trend in these resilience indicators before each collapse was determined as the Kendall’s τ rank correlation.

The significance of Kendall’s τ rank correlation can be looked up directly. However, since time-correlated data may produce spurious results we took a more careful bootstrap approach. For each of the time series we determined the probability of obtaining the Kendall τ by chance by comparing them with τ values determined using 1,000 randomized surrogate datasets of the same size. The surrogate datasets were generated by randomizing the phases of the power spectrum (59) of each of the original datasets. The estimated *P* values were determined as (n + 1)/1,000, where n is the number of times that the Kendall τ of the surrogate datasets exceeded the τ of the original dataset. We used Fisher’s combined probability test (60) to estimate the combined probability of the *P* values of the regimes by chance. We also explored the robustness of the results by testing the sensitivity of our Kendall τ values to the choice of the smoothing bandwidth and the size of the sliding window (*SI Appendix*, Figs. S5, S7, and S8).

To illustrate how loss of resilience of the status quo in a society may be detected from time series, we use a simple model of attitude toward the status quo (20, 34, 35):

$$A_{t}=\tanh\left(P+c\;A_{t-1}+\;\sigma \;\xi \left(t\right)\right).$$

Here *A*_*t*_ is the mean attitude toward the need for change at time *t*, depending on the perceived seriousness of problems (*P*) such as food insecurity or violence, and on social pressure (*c*) to adhere to the same attitude that is held by your peers. There is also a random factorσ ξ(t) that we may think of as the effect of climatic variability scaled by σ. As the perceived seriousness of problems increases the mean attitude first changes hardly at all due to the contagiousness of attitude, until the tipping point (F1 in panel *A*) is reached where the vast majority suddenly wants things to change. If we expose this model to random perturbations (σ>0) the attitude (*A*) fluctuates. One implication is that the shift to the alternative attitude will not happen precisely at the bifurcation point as the fluctuations can drive attitude across the critical saddle point for self-perpetuating change (the dashed unstable part of the equilibrium curve). More importantly, if we drive the model toward the tipping point by gradually increasing the perceived seriousness of the problems (*P*) the temporal autocorrelation (panel *C*) and variance (panel *D*) of the detrended fluctuations in attitude (panel *B*) rise prior to the transition. Those are generic indicators of critical slowing down, a phenomenon that is not specific to the simple model we use here for illustration but rather happens across a wide range of systems as they approach a tipping point (24, 27, 38). A deeper explanation of the model and an illustration of how it may produce cycles of growth and collapse is presented in *SI Appendix*.

## Supplementary Material

Supplementary File

## Data Availability

The compiled tree-ring dates from the Southwestern United States used in this study can be accessed at the Digital Archaeological Record at https://doi.org/10.6067/XCV82J6D7B (61). The precipitation reconstructions can be accessed at NOAA National Centers for Environmental Information (https://www.ncdc.noaa.gov/paleo-search/study/19783) (62). The MATLAB codes for generating the results can be found at https://git.wur.nl/sparcs/generic_ews-for-matlab/-/releases/v.1%252C0 (63).
