# Supplementary material for: Loss of resilience preceded transformations of pre-Hispanic Pueblo societies
Source: Proc Natl Acad Sci U S A. 2021 Apr 28;118(18):e2024397118. doi: 10.1073/pnas.2024397118 (PMC8106319; doi:10.1073/pnas.2024397118)
Supplement: Supplementary File [file pnas.2024397118.sapp.pdf]

Supplementary material

Loss of Resilience Preceded Transformation of Prehispanic Pueblo Societies

Marten Scheffer[1,*], Egbert H. van Nes [1], Darcy Bird [2], R. Kyle Bocinsky [3], and Timothy A. Kohler [2,3,4*]

[1] Environmental Science Department, Wageningen University, P.O. Box 47, 6700 AA Wageningen, The Netherlands; [2] Department of Anthropology, Washington State University, Pullman, WA 99164-4910; [3] Crow Canyon Archaeological Center, 23390 C R K, Cortez, CO 81321; [4] Santa Fe Institute, 1399 Hyde Park Rd., Santa Fe, NM 87501; [*] corresponding authors

Contents

I. A minimal model of attitude shifts 2

II. Results with 2-year smoothing 11

References 13

I. A minimal model of attitude shifts

To illustrate the phenomenon of critical slowing down, consider a minimal model of abandoning the status quo in a society. We define a variable attitude (*A*) which measures the average opinion and can range from -1 (acceptance of the status quo) to +1 (rejection). Note that the model is not meant to realistically describe what happens in the Pueblo societies, but merely to illustrate how observation of indicators of critical slowing down may reveal loss of resilience (1).

To see how we can set up such a model, imagine a society consisting of numerous individuals, each one somewhat different from the others. Now, for each person let the attitude towards the need for change be -1 if she is content with the status quo and +1 if she is discontent and wants things to change. All individuals determine their attitude towards the need for change depending on their own perceptions of the seriousness of problems (think for instance of food insecurity, unfair inequality or danger from uncontrolled violence). However, importantly they also have a tendency to copy the attitude of their peers which can lead to cascading change once a critical threshold is passed. We may capture the dynamics of the mean attitude across such a society in a simple stochastic difference equation, based on a common approach to describe shifts in opinion (2, 3):

$$A_t = \tanh(P + c\, A_{t-1} + \sigma\, \xi(t)) \qquad (1)$$

Where A_t is the mean attitude of people towards the need for change at time *t*, depending on the perceived seriousness of problems (*P*) such as food insecurity and violence, and the social pressure (*c*) to adhere to this same average attitude as in the previous time step (A_{t-1}). There is also a normally distributed random factor $\xi(t)$ scaled by σ. We may think of this as the effect of an external forcing such as the effect of climatic variability on harvests. The tanh function makes sure that attitude (A) remains between -1 and 1.

The basics of the model are simple. If peer pressure (*c*, the tendency to adhere to the same average attitude as in the previous time step) is small, the mean equilibrium attitude is a smooth function of the perceived seriousness of the problem, as gradually more and more individuals see the need for change. However, if the tendency to adhere to the same attitude is large this model predicts classical hysteresis and tipping points in attitude (Fig. S1). In this situation, as the perceived seriousness of the problems rises, the mean attitude first changes hardly at all due to the contagiousness of attitude, until the tipping point (a saddle-node bifurcation in technical terms) is reached where a shift to the alternative attitude (+1) occurs implying a situation where the vast majority suddenly wants things to change.

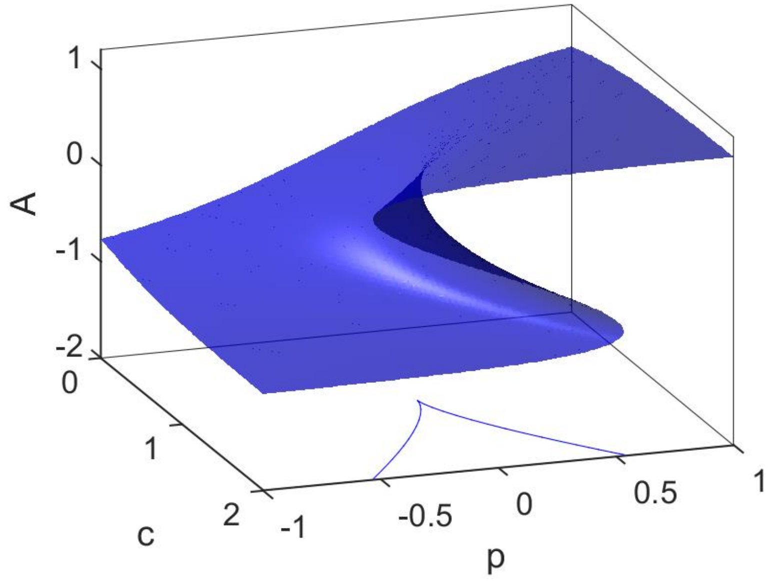

Figure S1. The equilibrium average attitude (A) to a problem as a function of perceived seriousness of the problem (P) and the social pressure to maintain the same attitude (c). The combinations of c and P where tipping points occur are shown below (blue line).

If we expose this model to noise, the attitude (*A*) always fluctuates. One implication is that the shift to the alternative attitude will not happen precisely at the bifurcation point as the fluctuations can drive attitude across the critical saddle-point for self-perpetuating change (the dashed unstable part of the equilibrium curve in panel A of the figure in box 1 of the main text). More importantly, the subtle nature of the fluctuations can tell us something about the resilience even far away from the shift.

If we drive the system towards the tipping point by gradually increasing the perceived seriousness of the problems (*P*) the temporal autocorrelation of the detrended fluctuations (the residuals presented in panel B of the box 1 figure in the main text) rises prior to the shift towards the alternative attitude (panel C in the figure in box 1 of the main text). This is the indicator of loss of resilience reflected in critical slowing down. On an intuitive level, one may think of this as resulting from the fact that the recovery rate from small perturbations goes to zero, thus leaving the state more correlated to the previous time step. Another way of framing the effect of critical slowing down is that the memory of the system increases. Thus, rising temporal autocorrelation is an indicator of decreasing resilience. Also, variance of the detrended fluctuations rises, which may be understood as an effect of critical slowing down too, as the decreased return rate to equilibrium causes the system to show a behavior that is more like an unconstrained random walk. While both autocorrelation and variance of detrended fluctuations may be affected by factors other than critical slowing down, the synchronous rise of those two indicators is considered a strong indication for a decrease of the intrinsic return rate to equilibrium and thus for a decrease of resilience. Note that those resilience indicators are not specific to the model we used here as an example. Critical slowing down occurs in a wide range of models and real-world systems as they approach a tipping point corresponding to a zero-eigenvalue bifurcation. Several overview articles give an accessible and extensive background on the nature and detection of those phenomena (1, 4, 5).

Cyclic transformations

The Pueblo societies we study go through a repeated series of transformations. To see how this may be mimicked by the model we assume that the perceived seriousness *P* increases gradually in time with speed γ_1. When the attitude shifts, we simply reset the system by letting *P* rapidly decline (with an arbitrary speed γ_2)

$$P_t = \begin{cases} P_{t-1} + \gamma_1 & if\ A_{t-1} < 0 \\ P_{t-1} - \gamma_2 & if\ A_{t-1} \geq 0 \end{cases} \quad (2)$$

We then applied our analysis of indicators of declining resilience to this model for illustration (Fig. S2). For this analysis, we simulated the model (equations 1and 2) 1000 times for 1500 years starting with a perceived seriousness P_t of -1 and an attitude A_t of -1 (Figure S2A). For each of these time series we extracted the increasing parts per cycle starting from attitude A_t of around -1 till just before the critical transition, which was defined as the time where a strong jump (by more than 0.5 units) in the attitude A_t occurred. This way, for each run of 1500 years, 4-5 segments of cycles were selected (total number of segments: 4047). For each of these segments we analyzed the indicators of resilience (see Section II of this Supplementary Information). The analysis of the Kendall's τ of the autocorrelation with time (Fig. S2b) shows that although the distribution of Kendall's τ is clearly different from the null model, there is a rather large probability of missing critical slowing down due to the limited number of data points (here ca. 300 points).

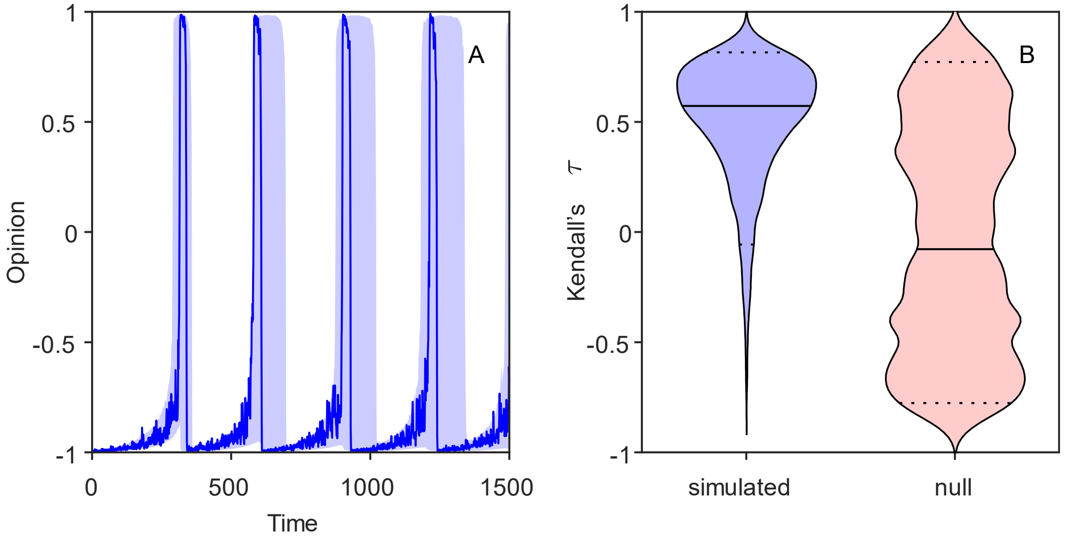

Figure S2. Statistical properties of our indicators of loss of resilience (see section II) illustrated on results of the cyclic version of the attitude model. Panel A: an example of a 1500-year run (the shaded area indicates the 5% and 95% percentiles of 1000 runs). Panel B: probability distribution of Kendall's rank correlation (τ) of the autocorrelation with time based on each increasing segment of cycles selected from 1000 runs of the cyclic opinion model (number of analyzed full cycles: 4047, number of data points per cycle ca. 300 points, size of the sliding window 50 % and bandwidth of smoothing 10 % of the data set) and the null model from which rising resilience is absent.

Table S1 Trends in temporal autocorrelation of tree-cutting activity for each of the periods (first two columns) and trends in cross-correlation between activity and maize niche size as climate indicator (3rd and 4th column) leading up to transformations. All these trends were determined in a moving window of 30 years (Early and Late PIII) or 60 years (all other cases). The significance was determined with a null model. The last two columns represent the overall correlation between activity and climate over each of the whole periods (rather than trends as in the previous columns). In the last two rows the combined p-values for indicated regimes are determined using Fisher's combined probability test.

Regime(s)	*Trend in AR construction activity*		*Trend in cross correlation Activity – climate*		*Mean cross correlation between Activity – climate*	
	Kendall-τ	*p-value*	*Kendall-τ*	*p-value*	*Kendall-τ*	*p-value*
BM III	*0.68*	*0.052*	*0.43*	*0.18*	*0.078*	*0.12*
P I	*0.61*	*0.082*	*0.46*	*0.17*	*0.087*	*0.10*
P II	*0.62*	*0.028*	*-0.18*	*0.67*	*0.15*	*0.0004*
Early P III	*0.70*	*0.031*	*0.45*	*0.15*	*0.15*	*0.08*
Late PIII	*-0.14*	*0.613*	*-0.26*	*0.72*	*0.13*	*0.13*
Whole PIII	*-0.78*	*0.927*	*-0.16*	*0.60*	*0.07*	*0.24*
BM III, P I, P II, Early + Late PIII	$\chi^2(10)=26.0$	*0.0038*	$\chi^2(10)=12.1$	*0.30*	$\chi^2(10)=33.5$	*0.0002*
BM III, P I, P II, Whole PIII	$\chi^2(8)=18.2$	*0.019*	$\chi^2(8)=8.3$	*0.37*	$\chi^2(8)=27.2$	*0.0006*

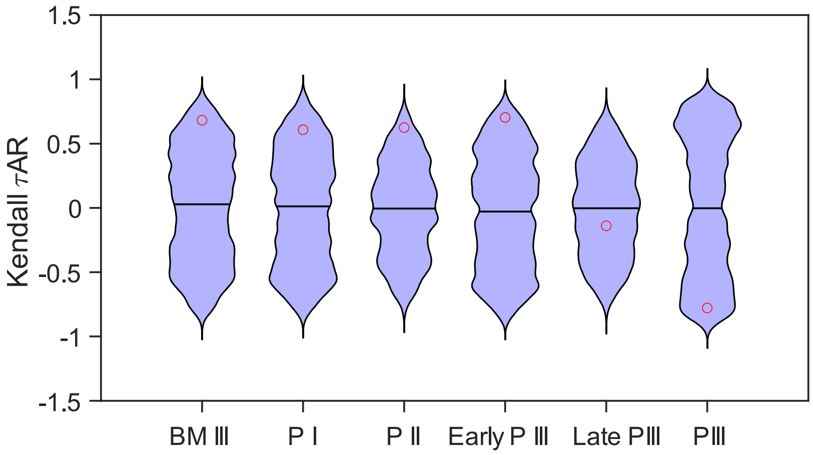

Figure S3. Violin plots of the Kendall τ values of the 1000 surrogate data generated for each of the periods. The actual τ value found in the original data is shown as red circle. Note how in the late PIII period the actual τ value is negative, signaling a decreasing trend in autocorrelation. This negative trend also prevails if the entire period III is analyzed using a large moving window (thus emphasizing the change towards the end of the period).

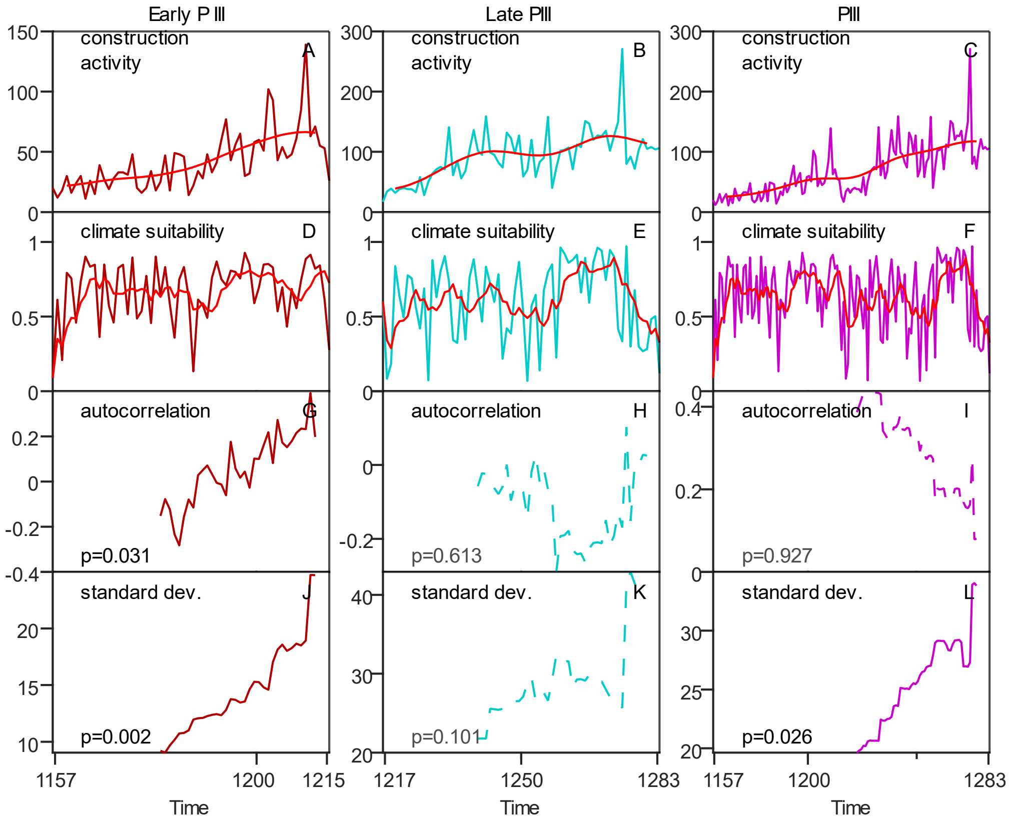

Figure S4. Patterns for construction activity, climate and resilience indicators for Pueblo III as well as its two sub-periods (variables as in Fig. 2 of the main text.). Over early PIII there is a steady rise of autocorrelation and variance in the construction activity, consistent with gradually declining resilience. This pattern (similar to what we see in the other periods) leads up to a sharp transition into late PIII, which is not recognized as one of the major transitions archeologically, but nonetheless corresponds to a marked change if we consider our proxy for construction activity. There is an abrupt drop (around 1218) in construction followed by about a decade of remarkably constant anomalously low activity. This early-late PIII transition is preceded by a few years with unusually bad climates for maize dry farming (the blue curve in the climate panel represents estimated maize productivity over the past two years, the red curve represents the mean over the preceding six years). The subsequent late PIII (unlike any other period) shows no significant trend in SD. Looking at PIII as a whole, it increases, but this due to computation over a longer window. AR-1 also initially stays roughly the same over late PIII, until from about the late 1250's it steeply declines, coinciding with a marked increase in climate conditions for maize production. Thus towards the end of (late) PIII we seem to see the signs of a society that becomes more resilient (as reflected in the fluctuations in construction activity) and over its last two decades experiences a steady and strong improvement of maize growing conditions. Note, however, that the subsequent decline in building activity (after 1274) does go hand in hand with worsening climate conditions for maize production (see Fig. 2 of the main text).

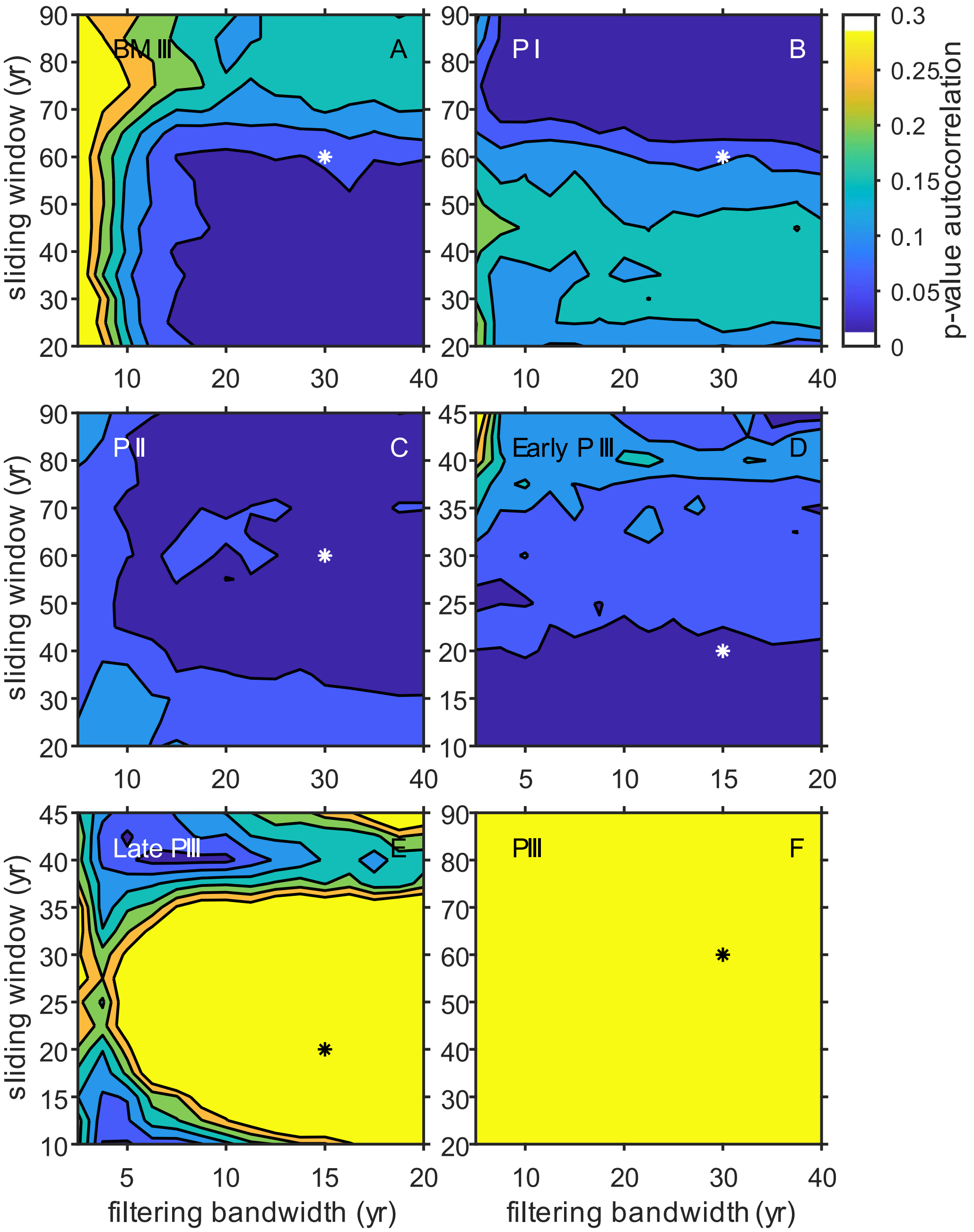

Figure S5. Sensitivity of the obtained Kendall-τ values to the sliding window and filtering bandwidth for the autocorrelation of the construction activity. The default bandwidth used for the results presented in the main text is indicated with an asterisk.

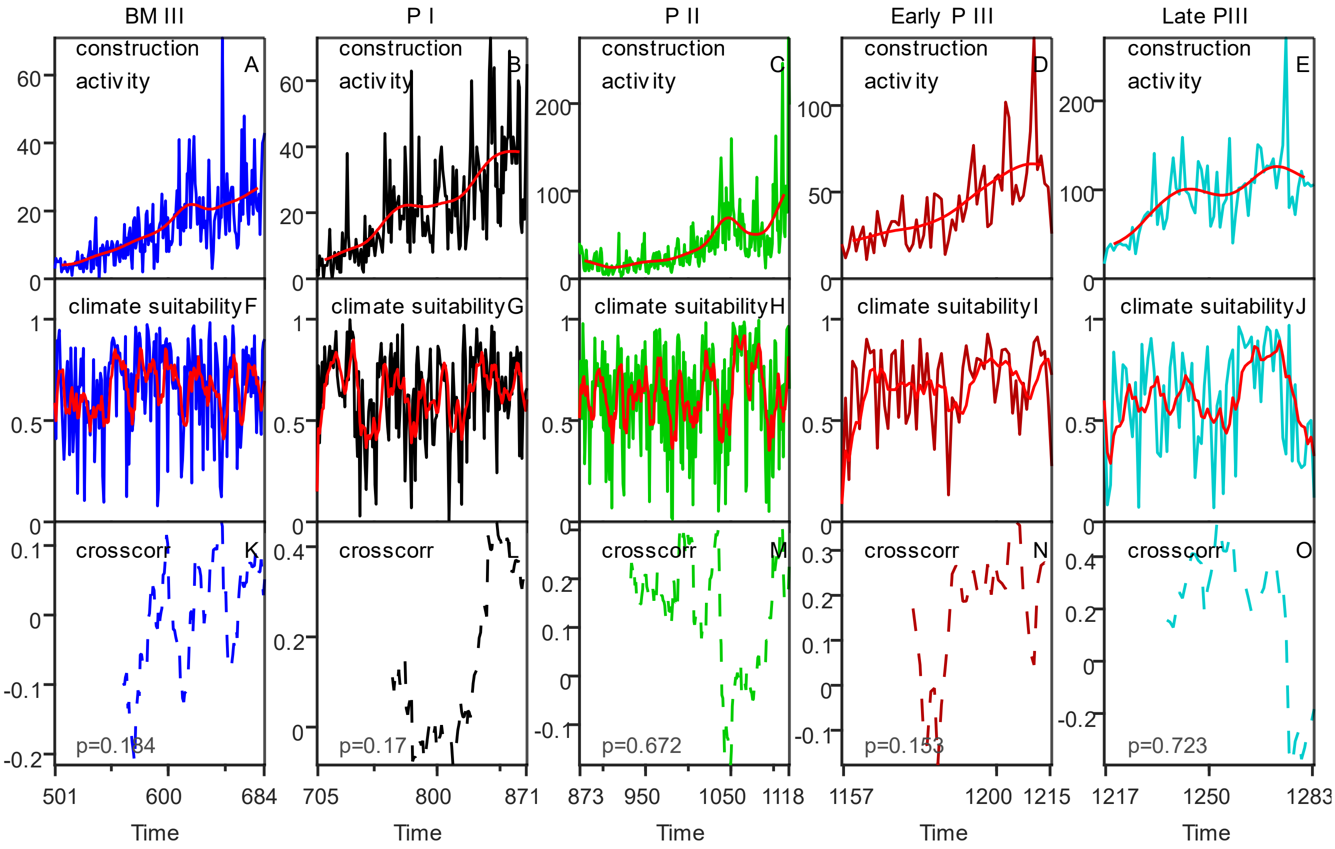

Figure S6. Relationship between construction activity and climate suitability. Panels A-J are also presented in the main text. Panels K-O show the variation over time of the cross correlation between construction activity and climate suitability for maize production. While those two variables tend to be positively correlated (better maize years correspond to higher construction activity), this correlation does not show consistent patterns over time across the periods.

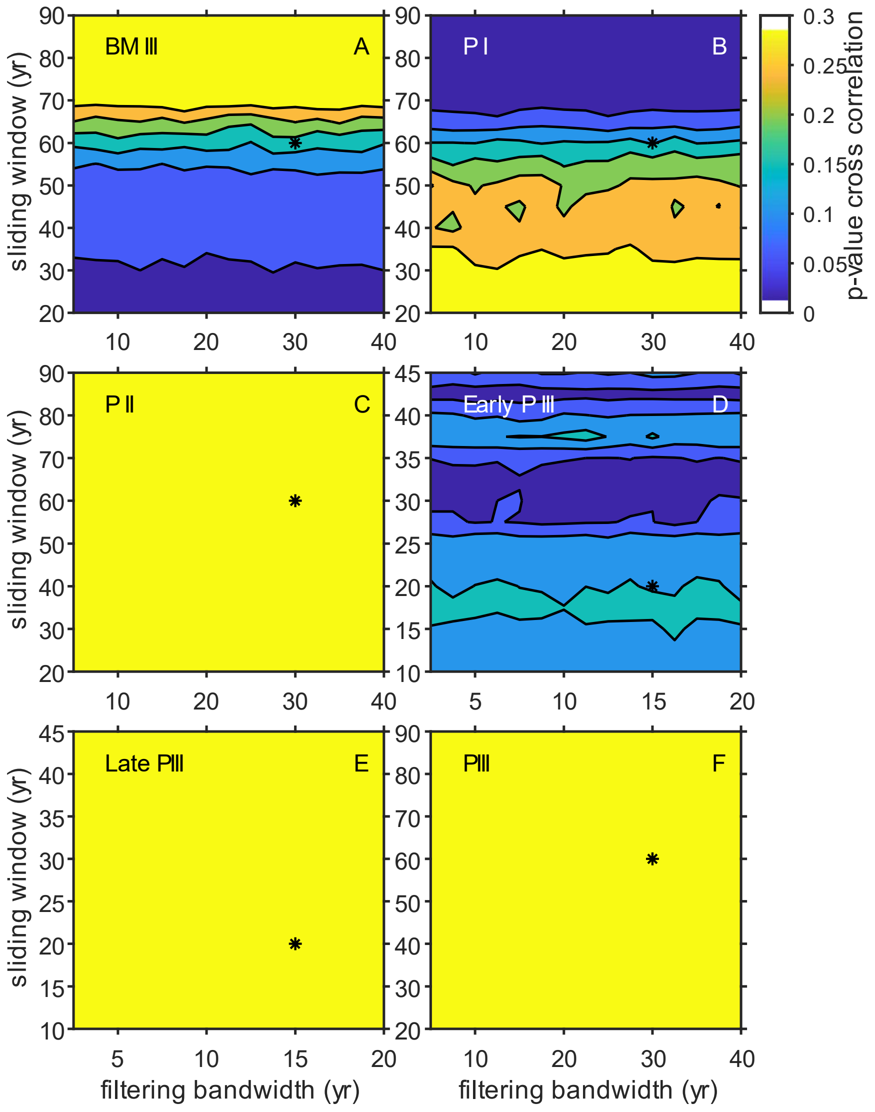

Figure S7. Sensitivity of the obtained p-values to the sliding window and filtering bandwidth for cross correlation. The default bandwidth used for the results presented in figure S6 is indicated with an asterisk.

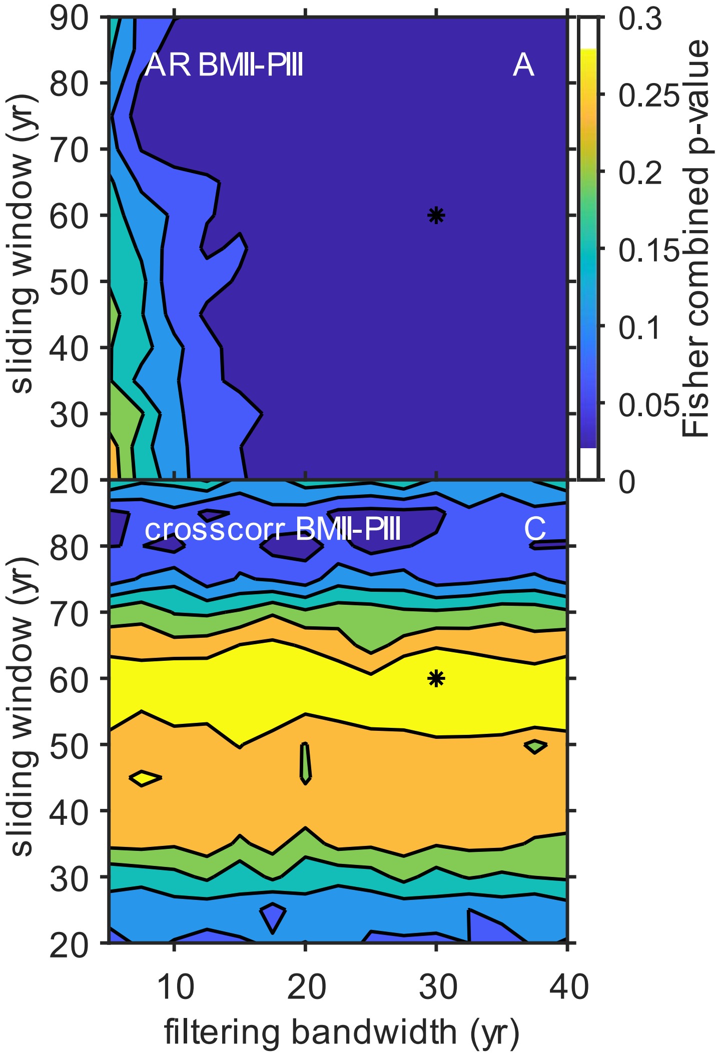

Figure S8. The sensitivity of the Fisher's combined p values for the filtering bandwidth and sliding window in the periods BM III, P I, P II, and the whole P III.

II. Results with 2-year smoothing

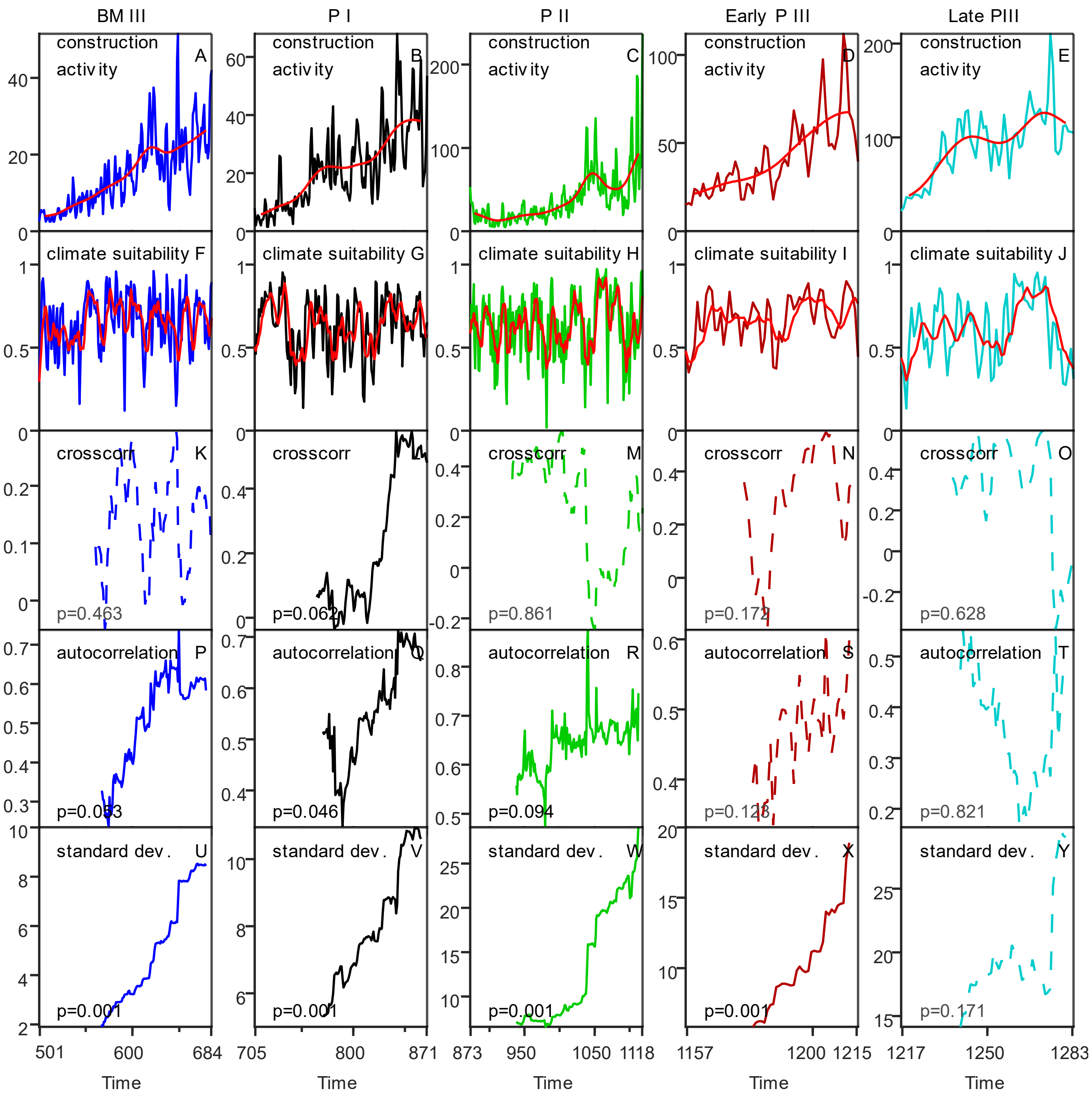

Figure S9. As Fig. 3 but with 2-year smoothing. The cross correlation as in Figure S6 with 2-year smoothing.

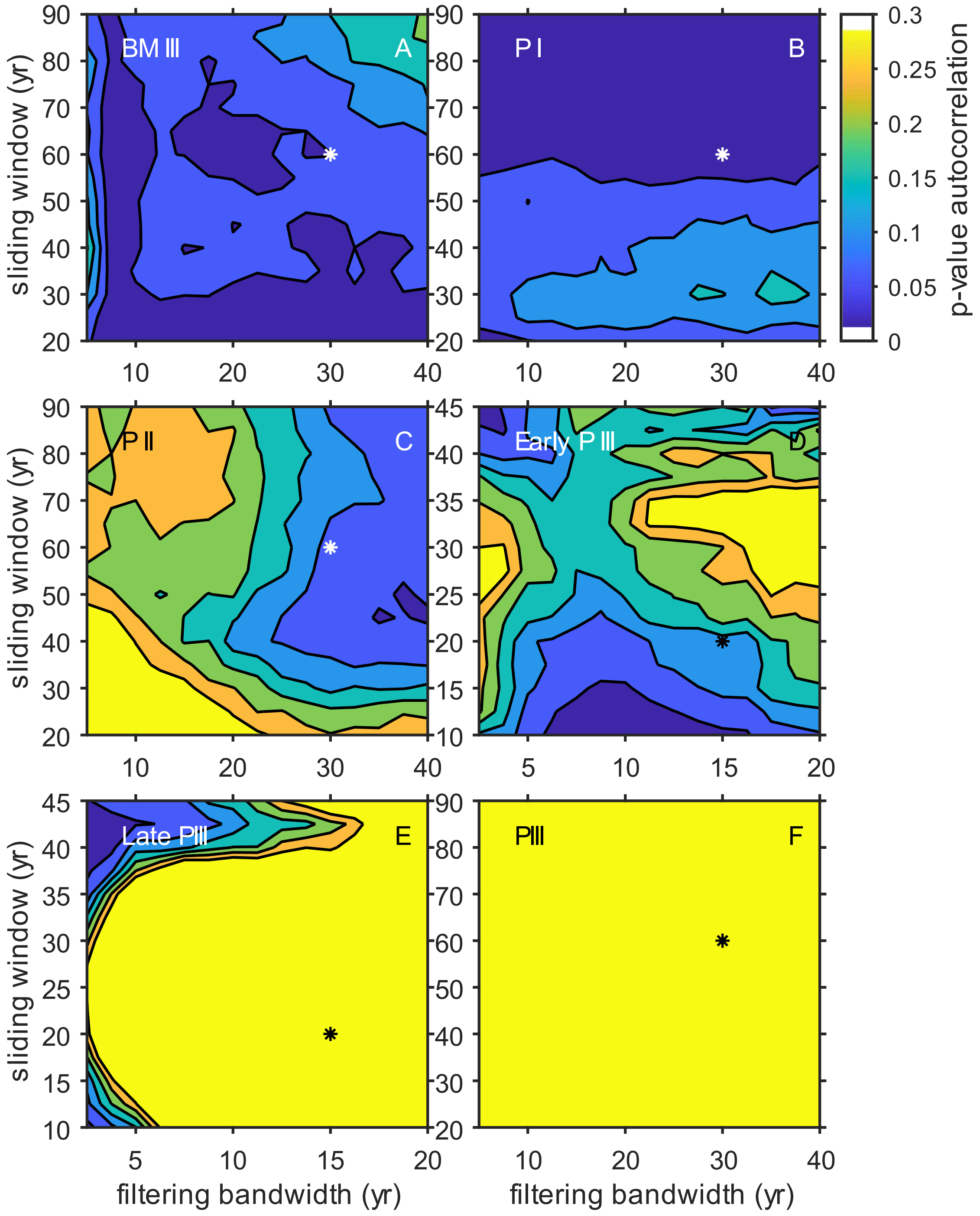

Figure S10. Sensitivity of the p-values for the bandwidth and window size as in Fig. S5 but with 2-year smoothing.

Table S2 the same as Table S1 but with 2-year smoothing

Regime(s)	*AR construction activity*		*Trend in cross correlation Activity - climate*		*Overall correlation Activity - climate*	
	Kendall-τ	*p-value*	*Kendall-τ*	*p-value*	*Kendall-τ*	*p-value*
BM III	*0.66*	*0.053*	*0.047*	*0.463*	*0.15*	*0.0027*
P I	*0.69*	*0.046*	*0.61*	*0.062*	*0.12*	*0.019*
P II	*0.50*	*0.094*	*-0.37*	*0.861*	*0.21*	*<0.00001*
Early P III	*0.49*	*0.12*	*0.4*	*0.172*	*0.30*	*0.00069*
Late PIII	*-0.33*	*0.82*	*-0.14*	*0.628*	*0.21*	*0.012*
Whole PIII	*-0.71*	*0.91*	*-0.26*	*0.663*	*0.12*	*0.051*
BM III, P I, P II, Early + Late PIII	$\chi 2(10)=21.5$	*0.019*	$\chi 2(10)=11.8$	*0.30*	$\chi 2\ (10)=70.5$	*<0.00001*
BM III, P I, P II, Whole PIII	$\chi 2(8)=17.0$	*0.03*	$\chi 2(8)=8.22$	*0.41*	$\chi 2\ (8)=53.0$	*<0.00001*

References

1. Scheffer M, *et al.* (2009) Early-warning signals for critical transitions. *Nature* 461(7260):53-59.
2. Brock W & Durlauf S (1999) A formal model of theory choice in science. *Economic Theory* 14:113-130.
3. Scheffer M, Westley F, & Brock W (2003) Slow response of societies to new problems: Causes and costs. *Ecosystems* 6(5):493-502.
4. Dakos V, *et al.* (2012) Methods for detecting early warnings of critical transitions in time series illustrated using simulated ecological data. *PLoS ONE* 7(7):e41010.
5. Scheffer M, Carpenter SR, Dakos V, & Van Nes EH (2015) Generic Indicators of Ecological Resilience: Inferring the Chance of a Critical Transition. *Annual Review of Ecology, Evolution, and Systematics* 46:145-167.
